# Development of a Simplified, Cost Effective GC-ECD Methodology for the Sensitive Detection of Bromoform in the Troposphere

**DOI:** 10.3390/s121013583

**Published:** 2012-10-10

**Authors:** Brett Kuyper, Casper Labuschagne, Raïssa Philibert, Nicholas Moyo, Howard Waldron, Chris Reason, Carl Palmer

**Affiliations:** 1 Department of Oceanography/MA-RE Institute, University of Cape Town, Rondebosch, 7701 Cape Town, South Africa; E-Mails: raissa.philibert@uct.ac.za (R.P.); nicholas.moyo@gmail.com (N.M.); howard.waldron@uct.ac.za (H.W.); chris.reason@uct.ac.za (C.R.); cpalmer@access.ac.za (C.P.); 2 South African Weather Services, CSIR, 7600 Stellenbosch, South Africa; E-Mail: casper.labuschagne@weathersa.co.za; 3 Applied Centre for Climate and Earth Systems Science, CHPC, Rosebank, 7700 Cape Town, South Africa

**Keywords:** bromoform, atmospheric chemistry, GC, ECD, VHOC, air samples

## Abstract

Wherever measurements have been made bromoform was found to be ubiquitous in the surface ocean in pmolar-nmolar concentrations. These measurements show concentrations in coastal regions orders of magnitude higher than in the pelagic oceans. Its atmospheric presence is primarily due to its release from algae and rapid transport to the marine boundary troposphere where it is known to participate in ozone chemistry via photochemical and catalytic pathways. Until quite recently, a limited number of studies existed (compared to other marine volatile organic compounds (VOCs)), mainly due to the analytical challenge(s) presented by the low environmental mixing ratios. In this work we detail the development of a simplified, cost effective method to detect and quantify bromoform in environmental air samples. Air samples (1.5 L) were preconcentrated onto a precooled adsorbent (Carbopack X/Carboxen 1016) trap. These samples were injected by means of rapid thermal desorption for separation and detection by GC-ECD. The system was calibrated by means of a custom-built permeation oven. A linear system response was achieved, having a detection limit of 0.73 ± 0.09 ppt. A range of environmental samples was analysed to demonstrate the ability of the technique to separate and identify bromoform from air samples. The results showed that bromoform concentrations typically averaged 24.7 ± 17.3 ppt in marine air samples, 68.5 ± 26.3 ppt in Cape Town urban air samples and 33.9 ± 40.5 ppt in simulated biomass burning plumes (SBBP).

## 1. Introduction

Bromoform is a naturally occurring organohalogen found in the marine boundary layer as a result of production by phytoplankton [1,2], ice algae [3] and kelp (e.g., [4–8]). The biological production of organohalogens is thought to be as an indirect consequence of oxidative stress [9]. Whereas there are some known anthropogenic sources of bromoform (e.g., water chlorination; coastal power generation) these sources are far smaller than natural sources when considered on a global scale [10]. Beingvolatile and not readily soluble in water bromoform readily crosses the sea-air boundary, resulting in significant (*ca.* ppt) concentrations in the marine troposphere [11]. There is some evidence to suggest a significant local terrestrial source of bromoform to the troposphere off the N. W. coast of Africa [12], but this source has neither been identified nor quantified.

Once in the marine boundary layer bromoform photolyses, (lifetime *ca.* 3 weeks) to produce bromine radicals, with two important implications for atmospheric chemistry: (i) bromine radical species can lead to catalytic boundary layer ozone depletion [13], thus perturbing the oxidative potential of the atmosphere; (ii) bromoform may persist long enough in the atmosphere to reach the stratosphere and thus contributes to stratospheric ozone depletion [14,15]. Recent modelling studies and measurements suggest that, due to rapid convection in the tropics, bromoform contributes significantly (30 to >60%) to inorganic bromine levels within the upper troposphere (UT)-lower stratosphere (LS) region [15–18]. This observation has significant implications for climate change, due to the interaction between bromine and stratospheric ozone found in the UT-LS region.

There have been a number of coastal fixed-station studies of bromoform in the northern hemisphere, most notably at Mace Head, Ireland [4], Kiel Fjord on the Baltic Sea [19] and the Canary Islands [20], but only one in the Southern Hemisphere, at Cape Grim, Tasmania [21]. These studies show concentrations in the atmosphere of up to 300 ppt [20]. A suite of cruise-based measurements also exists ([10] and references therein) showing lower concentrations (1–5 ppt) over the open Atlantic, Pacific and Southern Oceans (see the table in Section 3.3).

Compared to other biogenic atmospheric trace gases, such as dimethyl sulphate (DMS), there are still relatively few measurements of bromoform in any given marine environment and/or season. Cruise datasets that incorporate both ocean and air measurements have been used to estimate sea-air fluxes of bromoform [10]. However, these are too few and too irregular to extrapolate to an ocean basin or seasonal scale. A climatological approach based on the parameterisation of satellite data appears able to capture much of the known seasonal and spatial variation in this air-sea flux of bromoform [22]. However, this type of approach remains limited due to the lack of field data for validation. Furthermore, the magnitude of ozone destruction occurring in the troposphere as a result of this sea-air bromoform flux has been successfully modelled. The use of a parallel-tropospheric offline model of chemistry and transport has been extended to incorporate bromine chemistry [23]. This model in turn is only accurate if the boundary conditions, specifically the magnitude of the bromoform sea-air flux spatially and temporally, is well characterised. Thus, new field data in areas previously under-sampled are paramount to our understanding of tropospheric bromoform chemistry.

One of the most notable limitations of the current data is that there have been very few measurements made around southern Africa (none in the Indian Ocean), and no measurements made at fixed stations in sub-Saharan Africa. The paucity of data from southern Africa represents a significant gap in field measurements of bromoform. This is of specific concern as the highly biologically productive Benguela Current upwelling system could be a significant, hereto unquantified source of bromoform flux to the atmosphere.

From a marine perspective, the southern African region is of specific importance due to a wide range of marine habitats, which can be considered a natural laboratory for the comparison of bromoform production in widely differing marine environments. Southern Africa borders three diverse and globally significant ocean basins: the Indian, Atlantic and Southern Oceans. The geographical position of Cape Point in southwestern South Africa allows for the study of warm-ocean, rocky shores and ice-influenced waters all from one fixed point. Furthermore, it is situated within one of the world's most biologically productive upwelling systems, the Benguela, making it ideal to investigate the suggested link between bromoform and the observed high productivity [22].

From an atmospheric perspective South Africa is notable as is situated in the subtropics. The concentration of bromoform in the tropical troposphere is important, as it is in this region that rapid tropical convection allows bromoform to reach the upper troposphere and lower stratosphere [10].

South African institutions currently lack instrumental capacity to perform measurements of bromoform. In addition, there are very few South African scientists with the prerequisite skills to run an intensive monitoring programme should the infrastructure become available. Current bromoform measurements are, therefore, limited to irregular cruises conducted by visiting scientists. This situation fails to provide the global scientific community with comprehensive long-term and/or seasonal data sets or in transferring these skills to scientists of a developing country.

Here we present the development of a simple, cost effective instrument and methodology that will allow for long-term, sustained measurements of bromoform in southern Africa. This system is ideally suited for an African environment that does not always have first world resources necessary for expensive, sophisticated instrumentation.

## 2. Experimental Section

A Shimadzu GC-8 gas chromatograph (GC) was fitted with a J&W Scientific DB-624 (30 m × 0.32 mm × 1.8 μm, 5% polarity film) capillary column to achieve the optimum separation of bromoform. An electron capture detector (ECD) was used as the detector and maintained at 300 °C with 0.5 nA standing wave.

**Figure 1.**
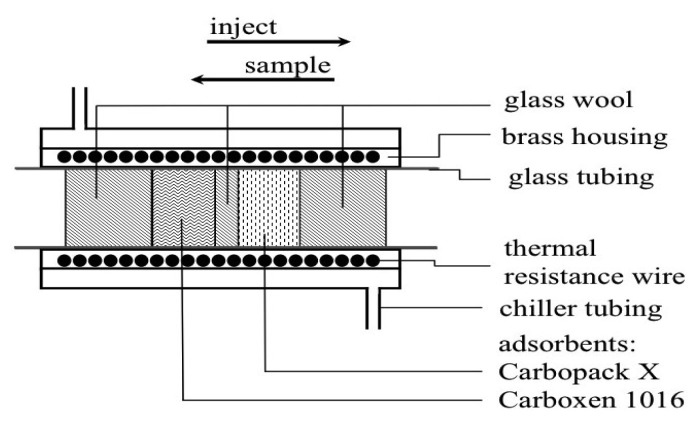
Schematic drawing showing the design of the custom-built thermal desorption unit. Samples were loaded onto 9 mg Carbopack X and 9 mg Carboxen 1016, passing the less adsorbent Carbopack X first. The unit was heated by means of high resistance thermal wire. During heating, the gas flow direction was reversed to inject the sample onto the column. Temperature within the unit was regulated using an imbedded Type K thermocouple.

The preconcentration of samples was achieved using a custom designed thermal desorption (TD) unit (UniTemp, Cape Town, South Africa). A brass tube containing a resistance heating wire housed within a brass water jacket. The jacket was cooled with a glycol solution at −15 ± 0.2 °C from a commercially available recirculating chiller (Figure 1). A glass tube housed 9 mg each of the two adsorbents (Carbopack X/Carboxen 1016) within the brass trap (Figure 1). These adsorbents preferentially remove and trap volatile halogenated organic compounds (VHOCs) from the air.

**Figure 2.**
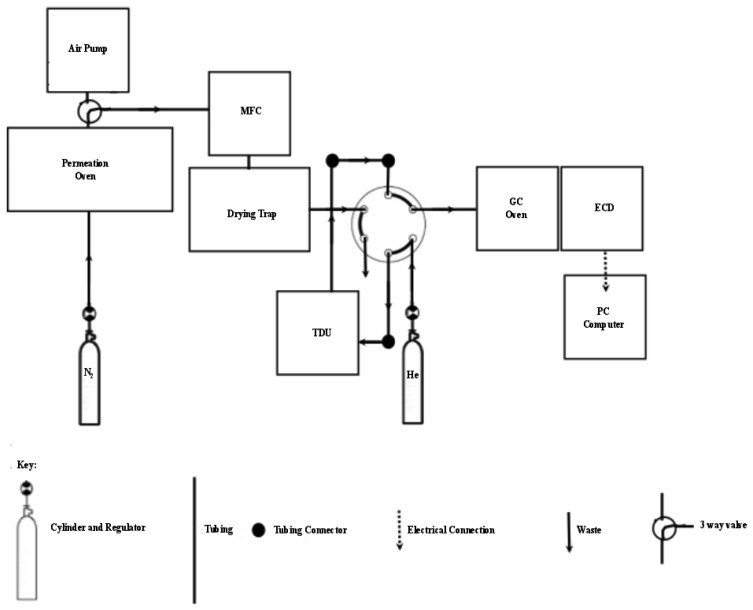
Schematic drawing showing the layout of the GC-ECD analysis system and associated gas and sample flows. Solid lines denote 1/8″ stainless steel tubing with the arrow denoting the direction of gas flow.

Switching between loading the sample onto the cooled trap and injecting the sample onto the column when the TD trap was heated was controlled by a six-port injection valve (Figure 2). A flow of 100 mL·min**^−^**^1^ through the trap was controlled with an ASM AFC-260 mass flow controller. Moisture was removed by magnesium perchlorate supported by plugs of glass wool in a small pipette [24]. The analogue ECD output was converted to a digital signal via a combination of Advantech ADAM 4017 and 4052 units and sent to a computer. MATLAB was used: (1) to develop a customised front end graphic user interface (GUI) for the real-time collection and display of the ECD signals, and (2) for the post run integration of chromatographic peaks.

### 2.1. Collection and Trapping of Sample onto Adsorbent Trap

Grade 5.0 nitrogen (Air Products/Air Liquide, Cape Town, South Africa) was introduced to the trap, replacing the air sample, in order to demonstrate that the system was able to produce a null result and that there was no carryover of bromoform from previous runs. The introduction of nitrogen was performed in the same manner as air or permeation gas, via a 3-valve (Figure 2), controlled through the mass flow controller at 100 mL·min**^−^**^1^ and passed through the six port injection valve set in the ‘load’ position and finally on to the adsorbent trap. The TD trap was maintained at −10 °C for the 15 min trapping period resulting in a 1.5 L sample volume. The procedure to ensure there was no carryover was performed on a daily basis, after each calibration point and every 5^th^ air sample.

### 2.2. Injection of Samples and GC Parameters

Once the samples were trapped on the absorbent tube they were immediately transferred to the column by switching the injection valve to the “inject” position and switching on the power supply resulting in rapid heating of the tube. The timing of the injection process was such that the oxygen peak was not flushed onto the GC column, after which the trap was maintained under a flow of helium (He) or nitrogen gas. Synchronisation of the injection was achieved by starting the heating of the trap followed by the rapid switching of the injection valve when the trap had reached a temperature of 15 °C. The trapped tube content was swept, by He carrier gas, onto the column for 1 min 20 s at which point the trap had reached ca. 300 °C. A constant 100 mL·min**^−^**^1^ nitrogen (N_2_) gas flow was maintained through the trap while it was cooled back to −10 °C ready for the next sample.

**Figure 3.**
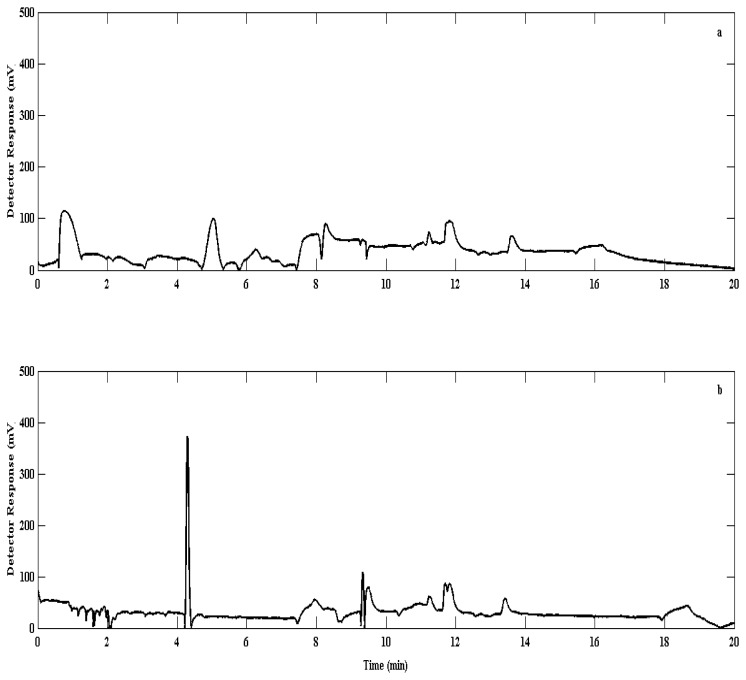
Chromatograms depicting the improvement of separation and chromatography by using liquid nitrogen [N_2(liq.)_] as a cryofocusing step. The chromatograms were recorded (**a**) without and (**b**) with the use of N_2(liq.)_ as a second cryofocusing stage. Note the difference in peak resolution at, for example, 5 min and better separation at 12 min. Bromoform is expected at just before 14 min.

The injection procedure resulted in relatively long injection time by the primary injector, thus requiring the need for second stage cryofocusing in order to produce high quality separation of elutants (Figure 3). This was introduced at the head of the column where the analyte was briefly captured in a secondary trap cooled to ca. −190 °C under liquid nitrogen. After trapping was finished desorption onto the column followed, by rapid heating to 100 °C using boiling water. It was found that this step improved both the separation of elutants and the sensitivity of the system.

**Figure 4.**
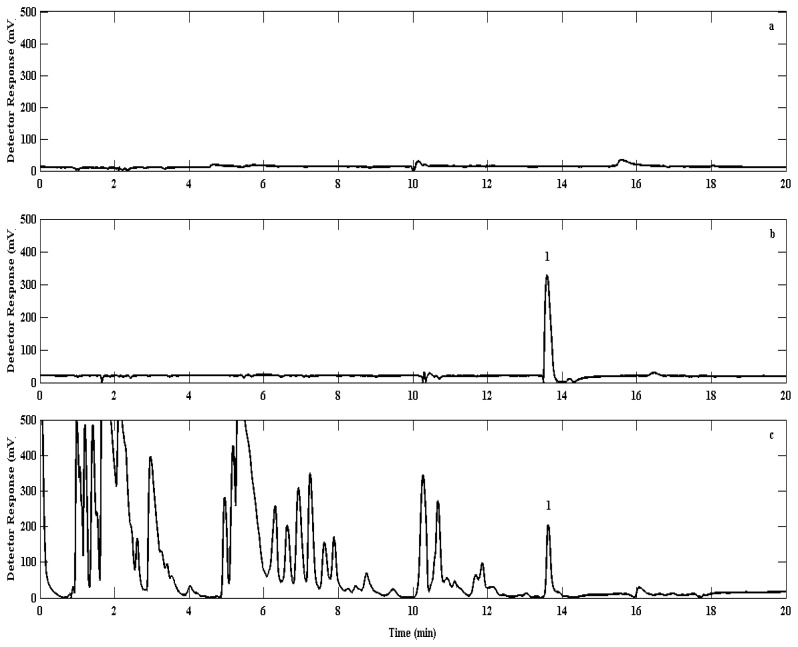
Chromatograms recorded on the GC-ECD system depicting a typical blank (**a**), 1× sampling loop of 100 μL calibration point (**b**) and a typical air sample (**c**), bromoform is identified as peak 1 in traces b and c.

The GC oven temperature was initially maintained at 30 °C during the injection and maintained for the first 5 min. Thereafter the oven temperature was ramped up every 5 min at a rate of 65 °C·min^−1^ to 60, 90, 150 and finally 200 °C. The last two temperature steps were required to ensure the complete removal of any contaminants from the column and system. When not stated, chromatograms were recorded with a constant backpressure of approx. 2 bar He (grade 5.0), resulting in a constant carrier gas flow of 5 mL·min^−1^. The carrier gas flow was complemented with make up gas (N_2_) set at 30 mL·min^−1^. Utilisation of this temperature programme and gas flow rate regime resulted in the suitable separation of bromoform from air samples for analysis (Figure 4).

### 2.3. Quantitative Trapping Tests

Samples of marine air were collected from a transect study across False Bay, about 20 km southeast from Cape Town CBD, aboard the *R.V. Sealab*. Samples were collected in electropolished stainless steel flasks using a metal bellows pump and a short section of PTFE tubing. For the purpose of these experiments, environmental samples rather than laboratory generated standards were chosen. Our reasoning included the fact that the target analyte for the work presented here was of marine origin, and realising that the breakthrough volume for specific chemical species may differ within a complex mixture cf. a single species chemical standard [25]. Quantitative trapping and breakthrough volume was verified by the passing of 1, 3 and 5 L samples of this collected air through two traps connected in series. The initial trap was maintained at room temperature (20–25 °C), whilst the secondary trap at −10 °C. After the first five minutes, the flow of air through the first trap was interrupted and the second tube, containing any breakthrough analytes within a 1 L sample, was heated using the thermal desorption apparatus and the sample was analysed using the GC-ECD as described earlier. This procedure was then repeated using the same primary tube, but a clean secondary tube resulting in an increasing volume trapped on the primary tube up to 5 L. The volume of the sample that had to be injected in order to be detectable on the secondary tube thus defines the breakthrough volume under these conditions.

### 2.4. Calibration

**Figure 5.**
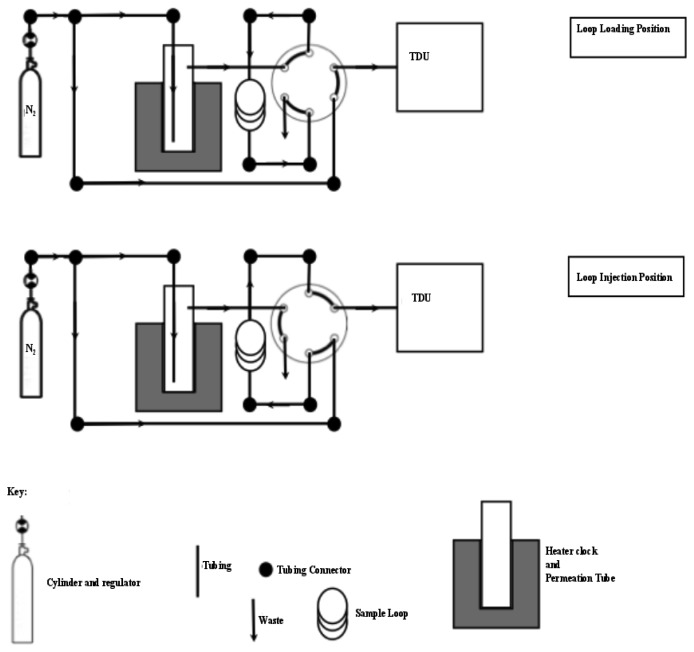
Schematic diagram illustrating the design and nitrogen gas flow of the permeation oven. Solid lines with arrows denote 1/8″ stainless steel tubing with the direction of N_2_ flow denoted by the arrow.

Calibration was done through a custom-built permeation oven (after Wevill and Carpenter [26]) (Figure 5) and a commercially purchased permeation tube (A&J Scientific, Cape Town, South Africa). The permeation tube was housed in an airtight gas bottle under a flow of 100 mL·min**^−^**^1^ N_2_ and was maintained at 70 °C. The exhaust from the bottle was continually passed through a calibrated 100 μL loop (Valco). A 6-port injection valve was used to inject a 100 μL volume of permeation gas onto the adsorbent trap by switching to the inject position (Figure 5) for 30 s. Repeated testing of differing times through the loop showed that 30 s was the optimal time required to completely flush the contents of the loop onto the TD trap. Repeated switching of the valve therefore allowed the injection of any multiple number of 100 μL loop volumes onto the cooled adsorbent trap. Calibration was achieved by triple repeat measurement of 1, 2 and 3 loop volumes in order to relate the recorded chromatographic peak area to the volume of permeation gas injected, as per Wevill and Carpenter [26]. Based on the flow rate through the airtight glass bottle and the known permeation rate of the tube (373 ng·min**^−^**^1^ at 70 °C), the concentration of bromoform in a loop can be calculated. The chromatographic peak area was related to the concentration through the number of moles of bromoform found in the sample loop. The mixing ratio is calculated based on the chromatographic peak area and an assumed air number density of 2.5 × 10^25^ molecules·m**^−^**^3^ [27].

### 2.5. Analysis of Environmental Samples

The ability of the method to identify and quantify bromoform in dilute environmental samples was tested on a varying range of conditions and locations. Specific experimental conditions and details of the sampling sites are given below:

#### 2.5.1. Cape Town Urban Air Samples

**Figure 6.**
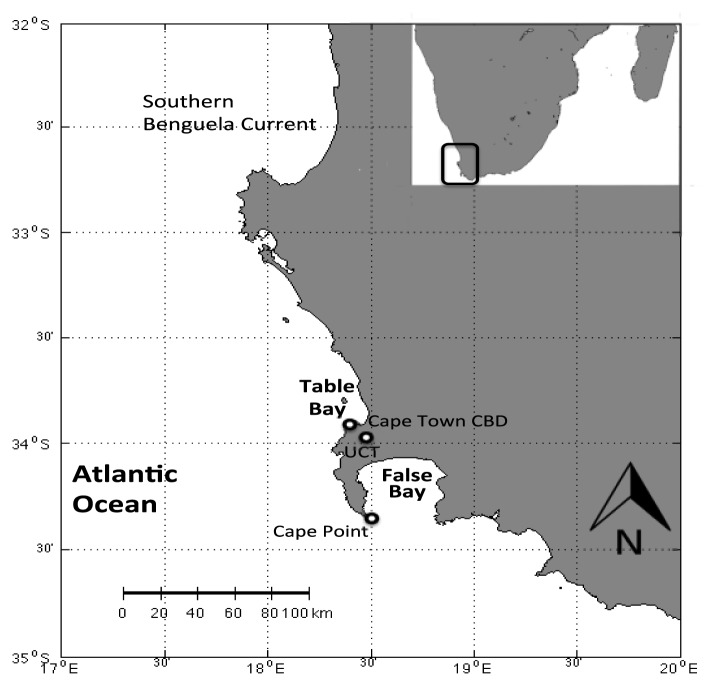
Map of the Cape Town area, highlighting the positions of Table and False Bays, the Benguela Current region. Cape Town CBD along with sampling sites at UCT and Cape Point are shown.

Air samples were collected outside the Department of Oceanography at the University of Cape Town (UCT; 33° 58′ S, 18° 28′ E/80 m ASL, Figure 6) in austral winter. The sample selection valve was set to the pump, and 1.5 L samples were drawn through a 5 m 1/8″ PTFE sampling line that was placed outside, approximately 1 m from paintwork to avoid contamination. Samples were trapped and injected as discussed earlier in the text. The sampling site is almost surrounded by oceans being about 10 km from Table Bay to the North and about 20 km from False Bay to the South East (Figure 6). This site is, however, characterised as being urban, *i.e.*, it is strongly affected by anthropogenic emissions such as from cars. The site located directly above Cape Town's southern suburbs, which spread to the South and East and within 10 km of the Northern Suburbs to the North East. The prevailing winds experienced at the site are from the North West in winter and from the South East in summer.

#### 2.5.2. Simulated Biomass Burning Plume (SBBP) Samples

A representative selection of local fynbos biomass was collected and within 3 h of collection placed in a concave steel bowl approximately 80 cm in diameter. The wood was then lit using kindling only, avoiding the use of any chemical firelighters. When the fire had ignited, a concave steel lid with a small sampling hole was placed over the bowl. The sampling line was then placed through the sampling hole and analysis was performed as per the method described for an air sample. The experiments were conducted within the Cape Town urban area at the location described in (*i*) during late austral winter/early spring. A series of tests was preformed using different mixtures of fynbos (mixture of wet and dry) and on different days.

#### 2.5.3. Cape Point Coastal Air Samples

Samples were collected at the Cape Point Global Atmospheric Watch (GAW) Station (34° 21′ S 18° 29′ E/230 m ASL, Figure 6) during austral spring.

Samples were drawn through 30 m inert Dekoron tubing by means of a grease free (*i.e.*, VOC free) pump. Apart from the change in sampling line the system setup was exactly the same as at UCT (Figure 2). Since these samples were collected in a high humidity environment, the air was dried in two stages using a cold trap followed by short stainless steel tube filled with magnesium perchlorate. Thereafter samples were trapped and analysed as described for air samples (*i*).

## 3. Results and Discussion

### 3.1. Breakthrough Analysis

**Figure 7.**
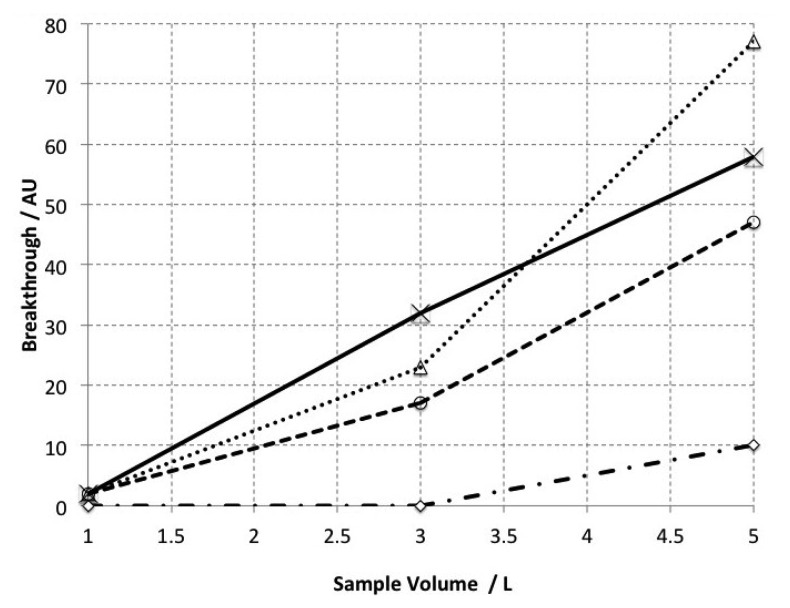
The amount of analyte breakthrough from the primary trap (as measured on the secondary trap) with increasing sample volume. The X-axis denotes the volume loaded onto the primary trap while the Y-axis denotes the cumulative peak area observed from analysing the secondary trap. Bromoform (t_R_ ca. 14 min) is denoted by dot-dash line. The other lines (dashed, dotted and solid) represent unidentified more volatile chemical constituents with t_R_ = 2.4, 2.7 and 3.0 respectively and is included here for comparative purposes.

Quantitative trapping tests showed no significant breakthrough of bromoform with sample volumes up to 3 L at ambient temperatures, whereas significant breakthrough occurred at 5 L (Figure 7). For more volatile compounds (unidentified but with constant retention times (t_R_) from 2–3 min), significant breakthrough was found to occur from 1 L. This implies that our method as described here is suitable for bromoform analysis within sample volumes of up to 3 L however; more work is needed to demonstrate whether this method is suitable for low molecular weight VHOCs.

### 3.2. Calibration and Standardisation

**Figure 8.**
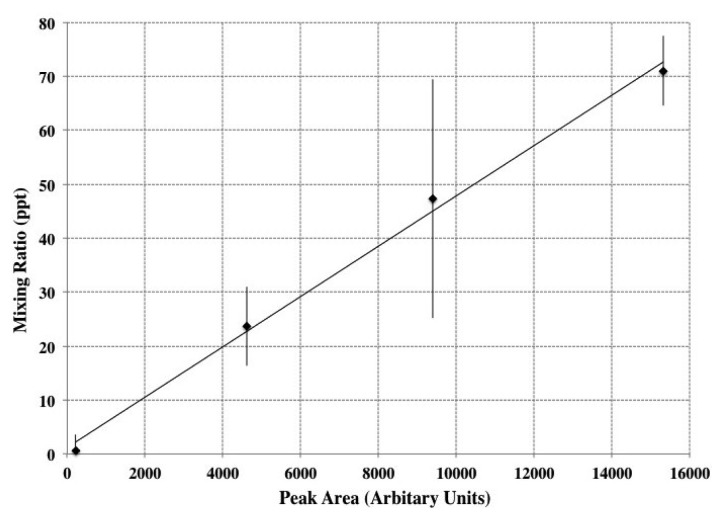
Calibration graph showing the correlation between the measured peak areas and bromoform mixing ratios (parts per trillion—ppt), error bars reflect the 95% confidence interval.

Calibration for bromoform showed that the system was linear within a range of concentrations of 5–90 ppt. Concentration *vs.* Peak Area gave R^2^ = 0.9 (Figure 8). The detection limit was determined to be 0.73 ± 0.09 ppt and the precision was found to 12.7% based on nine repeat 200 μL volume injections of bromoform standard.

#### 3.3. Environmental Samples

Injected nitrogen blanks showed no significant carryover from the previous sample analysis; the mean bromoform concentration in these experiments (0.06 ± 0.22 ppt) was observed to be an order of magnitude below the detection limit (0.73 ± 0.09 ppt). Mixing ratios of bromoform averaged 68.5 ± 26.3 ppt in urban air samples, with a maximum mixing ratio of 127.3 ± 16.2 ppt (Table 1). These data show significantly higher mixing ratios than Barletta *et al.*, [28] who reported urban concentrations in Karachi (Pakistan) to average about 2 ppt, with a maximum concentration around 7 ppt. The difference may be accounted for by the fact that although both cities are located next to an ocean, the Benguela is a much higher productivity ecosystem than the Arabian Sea. Furthermore, the Karachi study is averaged over several locations, some of which are about 15 km from the coast, whereas the work presented here represents just one location (UCT) 10 km from the coast (Figure 6). Thus, the differences between these two studies may be attributed to a number of factors notably differences in methods used, possible local anthropogenic sources such as water chlorination, different seasons, different oceanic and atmospheric conditions, differences in local and synoptic climatology for the two regions. The small number of samples taken in Cape Town urban air may be biased due to an extreme event and there may not be representative of the mean concentration.

Bromoform mixing ratios observed during the simulated biomass burning plumes (SBBP) averaged 33.9 ± 40.5 ppt. The Cape Town urban air showed an approximately double the SBBP mean value. Both data showed a high degree of variability with the SBBP data showing a larger range. Competition for adsorption sites within the trap and gas phase reactions in the burning plume could explain some of the variability within the SBBP dataset. However, the scope of experiment does not allow for firm conclusions to be drawn.

Mixing ratios were observed in marine air samples taken at Cape Point, with a maximum concentration of 84.7 ± 10.6 ppt. This is not surprising given that Cape Point is in close proximity to extensive kelp beds, and kelp being one of the primary sources of bromoform to the marine atmosphere. Northwesterly winds, which are most common in winter, bring air from the productive Benguela upwelling system, typified by the other major biological source, phytoplankton. The primary motivation for developing this system is the current paucity of data in the southern African region. For this very reason it is difficult to compare the result presented here with data from similar coastal upwelling sites. Such comparisons prove instructive as they serve to place the data within the context of past studies and also provide a first estimate of how this region may be different when compared to similar coastal measurement locations. The only Southern Hemisphere measurements at similar latitudes come from cruise measurements in the pelagic Indian Ocean. These show much lower concentrations than reported here, averaging 1.5 ppt and with a maximum reported of just 2 ppt. This is not surprising since it is well established that coastal concentrations of bromoform tend to be about 10–100 times higher than those over the open ocean [10,22]. Perhaps a more appropriate comparison is Cape Grim at 40° S in Tasmania; another rocky shore location with prevailing winds extending over a large open ocean fetch. However, the data presented here show bromoform mixing ratios that are of the order of five times higher than those observed at Cape Grim. A possible reason for this may be that Cape Point, unlike Cape Grim, is located adjacent to the southern Benguela, a highly productive upwelling region. In addition, Cape Grim is in a prevailing westerly wind regime throughout the year whereas this is only true of Cape Point during the winter. Cape Point is in close proximity to a large city that may have significant local anthropogenic sources that Cape Grim is free from, being in a more rural area. The most analogous system may be Gran Canaria (which also lies within a major upwelling system), which is at a similar latitude and longitude to Cape Point, but in the Northern Hemisphere. The atmospheric mixing ratios at Gran Canaria averaged 100 ppt [20], about five times higher than averages reported here. For the purposes of the discussion presented here, it is sufficient to note that the concentrations reported for Cape Point fall well within the range of data presented within literature. As noted, there are a variety of factors which may contribute to differences in observations. These factors and the reasons for the variability in bromoform concentrations observed at Cape Point are beyond the scope of this paper and will be discussed in more detail elsewhere [30].

## 4. Conclusions

We have documented the development of a simplified, cost effective methodology and system to detect and quantify the concentration of bromoform in environmental air samples. The method utilised a carbon-based adsorbent trap (Carbopack X/Carboxen 1016), which was cooled in two stages (−10 and −190 °C) for sample collection and rapidly heated to desorb analytes onto a GC column for separation. Bromoform was detected using an ECD and quantified using a custom-built permeation oven. Identification of bromoform was achieved based on retention time. The results show that using by this experimental design, 3 L air samples could be successfully preconcentrated with no significant breakthrough of bromoform. Calibration was linear within the range of concentrations to be studied and a detection limit of 0.73 ± 0.09 ppt was achieved with an overall precision of 12.7%. This method was developed to be able to sample a range of environmental mixtures. The data obtained showed a mean bromoform concentration of 68.5 ± 26.3 ppt in Cape Town urban air, approximately double the mean in a simulated biomass burning plume. Concentrations of bromoform measured at Cape Point Global Atmospheric Watch station were found to range from 2.29 to 84.7 ppt, with a mean value of 24.7 ± 17.3 ppt. While this is 5–7 times greater than most other studies, it does fall within the range of concentrations reported in the literature. Stations with a fairly similar marine environment such as Cape Grim and Mace Head, reported concentrations approximately 20 times lower than those in upwelling systems, like Gran Canaria. However, the Cape Point results are higher due to close proximity to both kelp beds and coastal upwelling and a possible anthropogenic influence. The method presented here provides a workable platform for future investigations to be carried out in this data sparse region.

## Figures and Tables

**Table 1.** Concentrations of bromoform in air samples from different locations around Cape Town compared to previously reported data. Concentrations are given in parts per trillion (ppt).

**Sample**	**Year**	**Latitude**	**Bromoform Concentration**				**Number of Samples**

			*mean*	*std. dev.*	*min.*	*max.*	
N_2_ Blanks *	2011	34 S	0.06	0.22	0	1.08	54
Cape Town Urban Air *	2011	34 S	68.5	26.3	50.6	127.3	7
Karachi Urban Air [28]	1988/1989	25 N	2.4	1.2	1.2	7.2	78
SBBP *	2011	34 S	33.9	40.5	2.5	158.2	19
Cape Point *	2011	34 S	24.7	17.3	2.29	84.7	131
Cape Grim [12]	1998	40 S	2.6	ND	0.7	8.0	>700
Mace Head [4]	1998	40 N	15	ND	10	23	>450
Gran Canaria [20]	1993	30 N	100	ND	25	300	ND
Indian Open Ocean [29]			1.5	ND	0.4	2	ND

* Data from this study, SBBP denotes simulated biomass burning plume experiments as described in Section 3.5.

## References

[1] Tokarczyk R., Moore R.M. (1994). Production of volatile organohalogens by phytoplankton cultures. Geophys. Res. Lett..

[2] Carpenter L.J., Wevill D.J., O'Doherty S., Spain G., Simmonds P.G. (2005). Atmospheric bromoform at Mace Head, Ireland: Seasonality evidence for a peatland source. Atmos. Chem. Phys..

[3] Sturges W.T., Cota G.F., Buckly P.T. (1992). Bromoform emissions from Arctic ice algae. Nature.

[4] Carpenter L.J., Malin G., Liss P.S., Kupper F.C. (2000). Novel biogenic iodine-containing trihalomethanes and other short lived halomethanes in the coastal East Atlantic. Global Biogeochem Cycles.

[5] Pedersen M., Collen J., Abrahamsson K., Ejdahl A. (1996). Production of halocarbons from seaweeds: An oxidation stress reaction?. Sci. Mar..

[6] Nightingale P.D., Malin G., Liss P.S. (1995). Production of chloroform and other low-molecular weight halocarbons by some species of macroalgae. Limnol. Oceanogr..

[7] Manley S.L., Goodwin K., North W.J. (1992). Laboratory production of bromoform, methylene bromide and methyl iodide by macroalgea and distribution in nearshore southern Californian waters. Limnol. Oceanogr..

[8] Gschwend P.M., MacFarlane J.K., Newman K.A. (1985). Volatile halogenated organic compounds released to seawater from temperate marine macroalgae. Science.

[9] Palmer C.J., Anders T.L., Carpenter L.J., Kupper F.C., McFiggans G.B. (2005). Iodine and halocarbon response of Laminaria Digitata to oxidative stress and links to atmospheric new particle production. Environ. Chem..

[10] Quack B., Wallace D.W.R. (2003). Air-sea flux of bromoform: Controls, rates and implications. Global Biogeochem Cycles.

[11] Carpenter L.J., Liss P.S. (2000). On temperate sources of bromoform and other reactive organic bromine gases. J. Geophys. Res..

[12] Carpenter L.J., Wevill D.J., Hopkins J.R., Dunk R.M., Jones C.E., Hornsby K.E., McQuaid J.B. (2007). Bromoform in tropical Atlantic air from 25° N to 25° S. Geophys. Res. Lett..

[13] Barrie L.A., Bottenheim J.W., Schnell R.C., Crutzen P.J., Rasmussen R.A. (1988). Ozone destruction and photochemical reactions at polar sunrise in the lower Antarctic atmosphere. Nature.

[14] Levine J.G., Braesicke P., Harris N.R.P., Savage N.H., Pyle J.A. (2007). Pathways and timescales for troposphere-to-stratosphere transport via the tropical tropopause layer and their relevance for very short lived substances. J. Geophys. Res..

[15] Sturges W.T., Glann C.F., Buckley P.T. (1997). Vertical profiles in snow, sea ice and seawater in the Canadian Arctic. J. Geophys. Res..

[16] Dvortsov V.L., Geller M.A., Solomon S., Schauffler S.M., Atlas E.L., Blake D.R. (1999). Rethinking reactive halogen budgets in the midlatitude lower stratosphere. Geophys. Res. Lett..

[17] Nielsen J.E., Douglass A.R. (2001). A simulation of bromoform's contribution to stratospheric bromine. J. Geophys. Res..

[18] Schauffler S.M., Atlas E., Flocke F., Lueb R.A., Stroud V., Travnicek W. (1998). Measurements of bromine containing organic compounds at the tropical tropopause. Geophys. Res. Lett..

[19] Fogelqvist E., Krysell M. (1991). Naturally and anthropogenically produced bromoform in the Kattegatt, a semi-enclosed oceanic basin. J. Atmos. Chem..

[20] Ekdahl A., Pedersen M., Abrahamsson K. (1998). A study of the diurnal variation of biogenic volatile halocarbons. Mar. Chem..

[21] Carpenter L.J., Liss P.S., Penkett S.A. (2003). Marine organohalogens in the atmosphere over the Atlantic and Southern Oceans. J. Geophys. Res..

[22] Palmer C.J., Reason C.J. (2009). Relationships of surface bromoform concentrations with mixed layer depth and salinity in the tropical oceans. Global Biogeochem Cycles.

[23] Yang X., Cox R.A., Warwick N.J., Pyle J.A., Carver G.D., O'Connor F.M., Savage N.H. (2005). Tropospheric bromine chemistry and its impacts on zone: A model study. J. Geophys. Res..

[24] Moore R.M., Zafiriou O.C. (1994). Photochemical production of methyl iodide in seawater. J. Geophys. Res..

[25] Palmer C.J. (2006). A study of the Distribution and Origin of Volatile Halogenated Organic Compounds in the Troposphere and the Oceans. Ph.D. Thesis.

[26] Wevill D.J., Carpenter L.J. (2004). Automated measurement and calibration of reactive volatile halogenated compounds in the atmosphere. Analyst.

[27] Nic M., Jirat J., Kosata B., McNaught A.D., Wilkinson A. (1997). IUPAC Compendium of Chemical Terminology.

[28] Barletta B., Meinardi S., Simpson I.J., Khwaja H.A., Blake D.R., Rowland F.S. (2002). Mixing ratios of volatile organic compounds (VOCs) in the atmosphere of Karachi, Pakistan. Atmos. Environ..

[29] Yokouchi Y., Nojiri Y., Barrie L.A., Toom-Sauntry D., Funjinuma Y. (2001). Atmospheric methyl iodide: High correlation with surface seawater and its implications on the sea to air flux. J. Geophys. Res..

[30] Kuyper B. An Investigation into the Source of Bromoform in the Southern African Marine Boundary Layer at Cape Point. Ph.D. Thesis.

